# Soil Moisture Retrieval in Farmland Areas with Sentinel Multi-Source Data Based on Regression Convolutional Neural Networks

**DOI:** 10.3390/s21030877

**Published:** 2021-01-28

**Authors:** Jian Liu, Youshuan Xu, Henghui Li, Jiao Guo

**Affiliations:** 1 College of Mechanical and Electronic Engineering, Northwest A&F University, Yangling 712100, China; echoliujian@nwsuaf.edu.cn (J.L.); li_henghui@nwafu.edu.cn (H.L.); 2 Shanghai Institute of Satellite Engineering, Shanghai 201109, China; xuyoushuan@163.com; 3 Shaanxi Key Laboratory of Agricultural Information Perception and Intelligent Service, Yangling 712100, China; 4 Key Laboratory of Agricultural Internet of Things, Ministry of Agriculture and Rural Affairs, Yangling 712100, China

**Keywords:** soil moisture content, farmland areas, sentinel, regression convolutional neural networks

## Abstract

As an important component of the earth ecosystem, soil moisture monitoring is of great significance in the fields of crop growth monitoring, crop yield estimation, variable irrigation, and other related applications. In order to mitigate or eliminate the impacts of sparse vegetation covers in farmland areas, this study combines multi-source remote sensing data from Sentinel-1 radar and Sentinel-2 optical satellites to quantitatively retrieve soil moisture content. Firstly, a traditional Oh model was applied to estimate soil moisture content after removing vegetation influence by a water cloud model. Secondly, support vector regression (SVR) and generalized regression neural network (GRNN) models were used to establish the relationships between various remote sensing features and real soil moisture. Finally, a regression convolutional neural network (CNNR) model is constructed to extract deep-level features of remote sensing data to increase soil moisture retrieval accuracy. In addition, polarimetric decomposition features for real Sentinel-1 PolSAR data are also included in the construction of inversion models. Based on the established soil moisture retrieval models, this study analyzes the influence of each input feature on the inversion accuracy in detail. The experimental results show that the optimal combination of *R*^2^ and root mean square error (RMSE) for SVR is 0.7619 and 0.0257 cm^3^/cm^3^, respectively. The optimal combination of *R*^2^ and RMSE for GRNN is 0.7098 and 0.0264 cm^3^/cm^3^, respectively. Especially, the CNNR model with optimal feature combination can generate inversion results with the highest accuracy, whose *R*^2^ and RMSE reach up to 0.8947 and 0.0208 cm^3^/cm^3^, respectively. Compared to other methods, the proposed algorithm improves the accuracy of soil moisture retrieval from synthetic aperture radar (SAR) and optical data. Furthermore, after adding polarization decomposition features, the *R*^2^ of CNNR is raised by 0.1524 and the RMSE of CNNR decreased by 0.0019 cm^3^/cm^3^ on average, which means that the addition of polarimetric decomposition features effectively improves the accuracy of soil moisture retrieval results.

## 1. Introduction

As an important component of the earth ecosystem, soil moisture content (SMC) is directly involved in the exchange of water and energy among surface water, groundwater, and atmospheric vapor [1,2]. Meanwhile, it also plays an active role in the fields of water resource management, ecological planning, agricultural production, and other related fields [3,4]. In agricultural applications, SMC is not only the basic condition for crop growth and development but also the key parameter for crop yield estimation, drought monitoring, and variable irrigation [5,6]. Consequently, it is of great significance to retrieve SMC accurately and in a timely manner.

SMC distribution is influenced by several interacting factors, such as soil characteristics, vegetation coverage, and climatic conditions. Therefore, it is relatively difficult to obtain large-scale SMC information efficiently with the traditional single-point measurement methods such as the drying method and digital probes [7,8]. Compared with ground single-point measurement methods, remote sensing technology has been gradually applied due to its wide coverage, strong timeliness, and low costs over the last three decades [9]. As the earliest and most mature technology of earth observation, optical remote sensing has always been playing an important role [10,11,12]. However, the optical features have reduced sensitivity to the water content of the observed target because the SMC retrieval is only based on indirect relationships, and the accuracy is in general low. Conversely, microwave remote sensing can directly obtain reflection information of SMC and has been widely used.

Synthetic aperture radar (SAR), as an active microwave remote sensing technique, has the capacity to penetrate surface vegetation, which is quite suitable for the quantitative inversion of farmland surface SMC. Consequently, there are many scientific methods and models for SMC retrieval with SAR data, such as the multi-temporal [13], empirical approaches [14,15], machine learning algorithms [16,17,18,19,20], change detection algorithms [21], and so on. In the area of bare soil, the backscattered radar signal is mainly in terms of SMC and soil roughness. However, if the surface is covered with vegetation, some additional measurements need to be applied to remove the vegetation influences on SAR backscatter coefficients. To deal with this problem, the optical and SAR data are combined, and several algorithms have been developed for estimation SMC [22,23]. Generally, these algorithms can be divided into two main categories: (1) algorithms that removed vegetation impact based on a vegetation microwave scattering model [24,25,26,27]; (2) algorithms in which vegetation impact was represented by vegetation indexes or polarization decomposition features and then the inversion model was employed to couple them with SMC [19,28,29].

For the former one, the vegetation backscattering could be effectively separated to obtain the backscattering information of the bare soil through the vegetation microwave scattering model, in which a water cloud model (WCM) is a commonly used model [24]. For example, Kong et al. [25] inverted farmland SMC using Radarsat-2 radar data and GF-1 optical data, and the results of SMC retrieval were improved after removing vegetation effects based on WCM. Sekertekin et al. [26] retrieved SMC over vegetation covered surfaces, and its results showed that WCM was effective at eliminating the effect of vegetation backscattering. Xing et al. [27] estimated the SMC using the Dubois model after removing crop contribution to radar backscattering by a modified WCM, and validation results presented satisfactory results. This approach is easy to use and can approximately mitigate the vegetation influences. However, the model ignores that the inhomogeneity of vegetation will produce some errors. In addition, the model parameters must be recalculated considering various vegetation conditions, which lead to an insufficient universality of the model.

For the latter one, vegetation impact was not individually removed. On the one hand, the impacts of vegetation cover were directly considered through the vegetation index. For example, Attarzadeh et al. [28] presented an approach for the retrieval of SMC by coupling single polarization C-band SAR and optical data at the plot scale in vegetated areas. The vegetation effect was represented by three groups indices that were extracted from the optical data. Holtgrave et al. [18] utilized Landsat-8 data to calculate the normalized vegetation index (NDVI) to compensate the effect of vegetation on radar backscattering and estimated SMC in vegetation covered flood plains with Sentinel-1 SAR data based on support vector regression. On the other hand, the phase information contained in SAR images was decomposed into polarimetric parameters to evaluate the vegetation impact. The H/A/*α* polarimetric decomposition (i.e., Cloude decomposition) model has been successfully employed in previous SMC retrieval [19,29]. For example, Özerdem et al. [19] employed the H/A/*α* method to decompose Radarsata-2 data to obtain the polarization characteristics inputted to the generalized regression neural network (GRNN) algorithm to retrieve SMC. The results indicated that entropy (H), anisotropy (A), and alpha angle (*α*) are helpful for improving the accuracy of SMC retrieval. Xie et al. [29] combined the H/A/*α* and Freeman Durden polarization decomposition methods for SMC retrieval from full-polarization Radarsat-2 data. These parameters are directly affected by soil moisture. Another advantage is that they can be calculated from satellite data without ground sampling. Therefore, in this study, the H/A/*α* decomposition model was applied for polarimetric scattering parameters.

More recently, convolutional neural networks (CNN) and deep learning theory have obtained considerable development for image classification [30] and surface parameter retrieval [31] in the remote sensing field. CNN could fully consider the spatial distribution of parameters, so it is usually employed for extracting deeper features. These features are also gradually applied for SMC regression analysis with good accuracy and applicability [32].

Furthermore, CNN has the capability of fusing optical and radar data and is therefore a well adapted tool for extracting features. Unfortunately, there are few studies on exploiting the potential of combining SAR data with CNN to retrieve SMC; this study aims to construct a regression convolutional neural network (CNNR) for SMC retrieval. Notably, SMC retrieval factors usually include the backscattering coefficients (such as VV, VH) of active microwave radar data and vegetation factors (such as normalized difference vegetation index (NDVI), modified soil adjusted vegetation index (MSAVI)) calculated from optical data. Little consideration is given to the phase information of SAR data, which actually affects the accuracy of SMC retrieval. So, this study first employed an Oh model to retrieve SMC with a water cloud model and then evaluated the applicability of SVR and GRNN models. CNNR also was employed to extract the inner feature in satellite data that involves the strength and phase information of radar data and vegetation information of optical data. Finally, CNNR was constructed to improve the prediction performance, and the conclusions are presented in the last section.

The remaining sections are arranged as follows. In Section 2, the retrieval algorithms are introduced. Section 3 introduces the study area, ground truth data collection, and remote sensing datasets used in this paper. In Section 4, the performances of the constructed models are assessed. The importance of various features is also analyzed in this section. Section 5 discusses the advantages of the proposed method and the limitations of this work. In the end, Section 6 concludes this paper.

## 2. Methods

**Figure 1:**
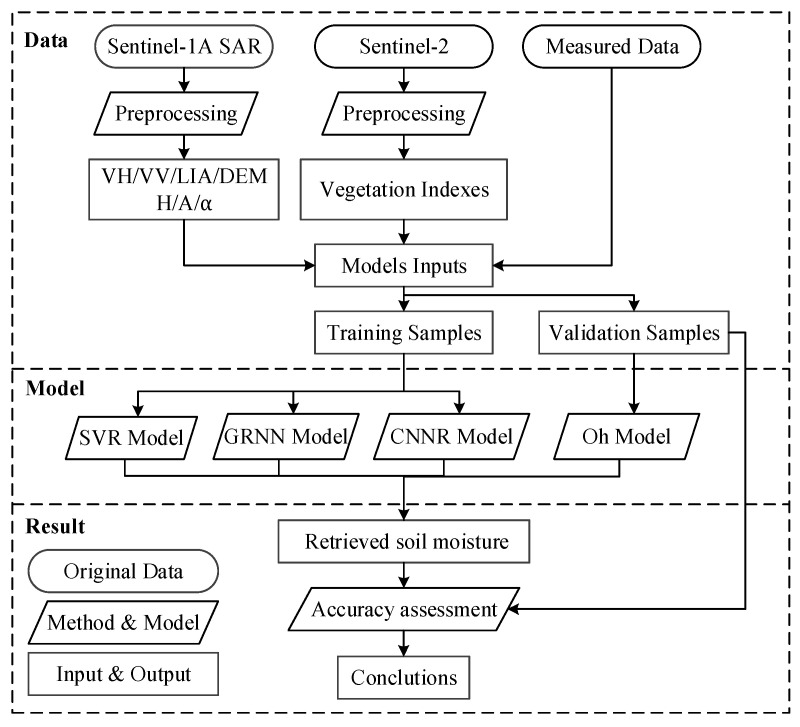
Specific flow chart of the research.

The model inputs of this paper include the backscattering coefficient of dual polarization radar (VH, VV), elevation and local incidence angle (LIA), vegetation indexes (NDVI, MSAVI, difference vegetation index (DVI)) and polarization decomposition features (H, A, *α*). The specific flow chart of the research was demonstrated in Figure 1.

### 2.1. Oh Model and Water Cloud Model

Many backscattering models have been developed to explain the relation between the SMC and SAR data; the Oh model was one of the most widely applied among these backscattering models. The empirical expressions of the Oh model are written as follows [33]:

(1) $$\sigma_{vh}^{0}=0.11m_{v}^{0.7}{\left(\cos\theta \right)}^{2.2}[1-\exp(-0.32{\left(ks\right)}^{1.8})]$$

(2) $$p=\frac{\sigma_{hh}^{0}}{\sigma_{vv}^{0}}=1-{\left(\frac{2\theta }{\pi }\right)}^{0.35m_{v}^{-0.65}}\cdot \exp(-0.4{\left(ks\right)}^{1.4})$$

(3) $$q=\frac{\sigma_{vh}^{0}}{\sigma_{vv}^{0}}=0.095{\left(0.13+\sin1.5\theta \right)}^{1.4}(1-\exp(-1.3{\left(ks\right)}^{0.9}))$$

where *p* and *q* represent the co-polarized ratio and the cross-polarized ratio, respectively, *θ* is the radar wave incident angle, *k* is the wave number (*k* = 2π/*λ* where *λ* is the wavelength), and *s* is the standard deviation of surface height. As the Sentinel-1 provides the backscattering coefficients in VH and VV polarizations, only Formulas (1) and (3) are employed to retrieve SMC. In addition, the study area in this paper was sparsely covered by winter wheat during the data acquisition time, and the backscatter coefficients extracted from radar images included the influence of soil surface vegetation, so the water cloud model (WCM) was used to separate the backscatter coefficients of vegetation. The WCM is shown as follows:

(4) $$\sigma_{can}^{0}(\theta )=\sigma_{veg}^{0}(\theta )+\tau^{2}(\theta )\cdot \sigma_{soil}^{0}(\theta )$$

(5) $$\sigma_{veg}^{0}(\theta )=A\times \mathrm{VWC}\cos\theta (1-\tau^{2}(\theta ))$$

(6) $$\tau^{2}(\theta )=\exp(-2B\times \mathrm{VWC}\sec\theta )$$

where $\sigma_{can}^{0}\left(\theta \right)$ is the total backscattering coefficient of the vegetated surface, $\sigma_{veg}^{0}\left(\theta \right)$ is the direct backscattering coefficient of the vegetation layer, $\sigma_{soil}^{0}\left(\theta \right)$ is the direct backscattering coefficient of the soil surface, and $\tau^{2}\left(\theta \right)$ is the two-way transmissivity of the vegetation layer. VWC is the vegetation water content (kg/m^2^), *θ* is the radar wave incident angle, and A and B are empirical parameters depending on the vegetation type. The vegetation type in this study area was mainly winter wheat. According to the literature [34], A and B in WCM were taken 0.0018 and 0.138, respectively. In consideration of the relationship between NDVI and VWC [25,35], VWC could be expressed as follows:

(7) VWC=0(NDVI≤0.17)

(8) VWC=4.285 7NDVI−1.542 9(NDVI>0.5)

(9) $$\mathrm{VWC}=1.913\;4{\mathrm{NDVI}}^{2}-0.321\;5\mathrm{NDVI}(0.17<\mathrm{NDVI}\leq 0.5).$$

Finally, the soil backscatter coefficient could be separated based on Formulas (4)–(9).

### 2.2. Support Vector Regression

Support vector regression (SVR) is the application of support vector machine (SVM) in function regression fitting based on the theory of minimizing structural risk. With the ascendancies of strong generalization ability and the ability to work well with both large and small samples, SVR is widely utilized to extract surface parameters using satellite imagery, such as soil salinity mapping [36], SMC retrieval [26], and crop yield prediction [37].

When kernel function *k*(*xi*, *x*) satisfies Mercer’s theorem, the corresponding regression prediction function could be described as Formula (10) according to the related theory of universal functions,

(10) $$f(x)=\sum_{i=1}^{n}\left(\alpha_{i}-\alpha_{i}^{*}\right)\cdot k(x_{i},x)+b$$

where *n* is the number of training samples, *α_i_*, α_i_* represent the Lagrange operator, and b is the bias. The Gaussian radial basis function was selected as the kernel function of SVR in this study. The best combination of the penalty factor *c* and parameter *g* are determined by the K-fold cross-validation method (K = 5), and the input data would be normalized uniformly, since the radiation values of optical data and radar data are quite different.

### 2.3. Generalized Regression Neural Network

Generalized regression neural network (GRNN) is a kind of local approximation network with radial basis kernel function composed of input, pattern, summation, and output layer [38]. The main advantages of this network are that it is suitable for solving nonlinear problems and has a low requirement for sample size, so it has been diffusely made use of in meteorology [39], food inspection [40], and other fields. In the GRNN model, the target parameter *Y*(*x*) is calculated as

(11) $$Y(x)=\frac{\sum_{i=1}^{n}y_{i}\exp[-{\left(x-x_{i}\right)}^{T}\cdot (x-x_{i})/2\sigma^{2}]}{\sum_{i=1}^{n}\exp[-{\left(x-x_{i}\right)}^{T}\cdot (x-x_{i})/2\sigma^{2}]}$$

where *n* is the sample size, *x* represents the measured input value, and *x_i_* and *y_i_* are the *i*-th neuron of the corresponding sample and the *i*-th observed value, respectively. As indicated in Formula (11), the performance of GRNN is greatly affected by its smoothing factor σ.

### 2.4. Regression Convolutional Neural Network

**Figure 2:**
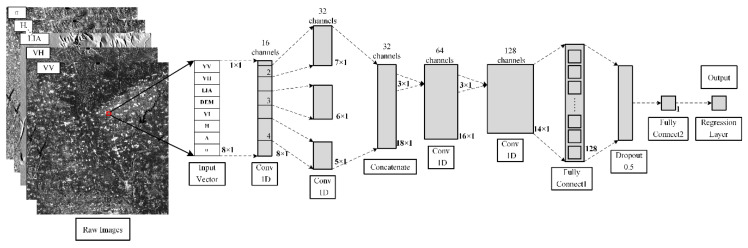
Structure of regression convolutional neural network (CNNR) in this study.

CNN proposed by Lecun et al. in 1998 has the most outstanding performance in image recognition [41,42]. The structure of a typical CNN network is consisted of convolutional, pooling, excitation function, fully connected layers, and a classifier. In order to avoid the reduction of inversion accuracy caused by decreasing feature information, the pooling layer in the CNN is removed from the model. Meanwhile, the classifier in the last layer of CNN is replaced by a regressor. The ReLU was selected as the excitation function with the advantage that ReLU is an unsaturated nonlinear function, and there is no problem of gradient disappearance [43]. For non-polarization decomposition feature combinations, the input characteristic parameters include $\sigma_{VH}^{0}$, $\sigma_{VV}^{0}$, DEM, LIA, and one of the three vegetation indexes, creating a 5 × 1 one-dimensional vector. For polarization decomposition feature combinations, the input characteristic parameters include $\sigma_{VH}^{0}$, $\sigma_{VV}^{0}$, DEM, LIA, H, A, *α*, and one of the three vegetation indexes, creating an 8 × 1 one-dimensional vector. Since the data size would be changed after adding the polarization decomposition feature, the input of non-polarization decomposition feature combinations is uniformly expanded into an 8 × 1 vector for modeling to facilitate comparison. The prediction accuracy of CNN is highly affected by the number and size of convolution kernels and its network structure. After repeated experiments using the existing data, the final structure is shown in Figure 2. The optimized Conv1D model mainly includes four Conv1D layers and two fully connected layers. The former two Conv1D layers were used to expand the depth of the data, and the latter two Conv1D layers were utilized to extract deep features. The output of the second Conv1D layers is a concatenation of a width 2 Conv1D layer, a width 3 Conv1D layer, and a width 4 Conv1D layer. In addition, the ReLU layer is connected after each convolution layer. Two fully connected and dropout layers were set up to raise regression fitting capability.

## 3. Materials

### 3.1. Study Area

**Figure 3:**
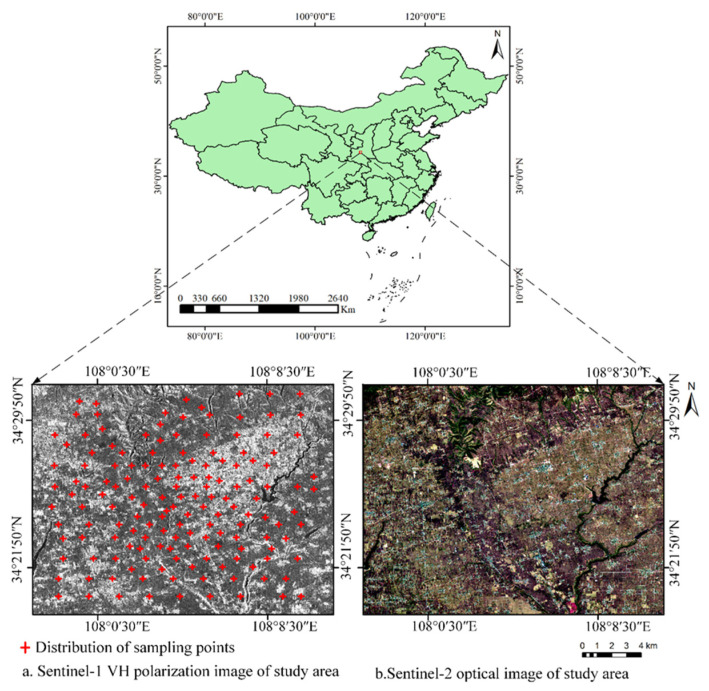
The location of the study area, shown as a Sentinel-1A SAR image from March 2018 (**a**) and the Sentinel-2A optical image from March 2018 (**b**).

An agricultural region in the Yangling agricultural hi-tech industries demonstration zone, one of the districts of Shaanxi province, China, was chosen as the study area (Figure 3). The study area has a spatial extent of approximately 20 km × 16 km (107°55′20″ E~108°15′40″ E, 34°15′15″ N~34°50′28″ N) and is located in the middle of Guanzhong Plain, which is a dominant wheat-producing area in China. The terrain in this area is relatively flat with an altitude between 560 and 790 m. The jointing period of wheat grows after April, so during the investigated period, the farmland surface was bare or covered by little vegetation. Figure 3a,b show the Sentinel-1 SAR image of VH polarization mode and Sentinel-2 optical image, respectively.

### 3.2. Ground Measurements

The ground measurements were collected during 12 March 2018, 8 December 2019, and 6 January 2020. Field sampling times were as consistent as possible with the time of the satellite overpasses to reduce measurement errors. SMC were obtained using a TDR-300 SMC meter, whose probe length is 5 cm, which is suitable for acquiring surface SMC. For each sample, five replicate measurements of SMC were recorded and averaged. To ensure the authenticity of ground sampling, these soil samples were placed in a 100 cm^3^ aluminum box and then dried to calculate SMC. The latitude and longitude of each sample were received by a handheld global positioning system (GPS) with an accuracy of cm, which is much smaller than the resolution of remote sensing images. After excluding the abnormal points, the remaining 154 samples totaled in the three samplings are further studied, and the statistics of the SMC for each period are shown in Table 1. The sample set partitioning based on joint x-y distances (SPXY) were applied to select the training and testing dataset [44]. In total, 123 samples in the measured data are used as the training set of these models, and 31 remaining samples are adopted as validation set.

### 3.3. Remote Sensing Data

Sentinel-1 radar and Setinel-2 optical satellite belong to the Sentinel series of satellites developed by the European Space Agency (ESA), which is designed to dynamically monitor the environment and safety of the earth. Sentinel-1 is equipped with C-band with advantages of dual polarization and short time resolution. Ground Range Detected (GRD) and three Single Look Complex (SLC) format data in Interferometric Wide Swath (IW) mode were selected in this study. The multi-spectral imager installed on the Sentinel-2 has 13 bands ranging from visible light to short-wave infrared with different spatial resolutions. These data could be downloaded freely from the website (https://scihub.copernicus.eu), and more characteristics of the applied remote sensing data were introduced in Table 2.

The preprocessing of Sentinel images is mainly carried out on the corresponding software (Sentinel Application Platform, SNAP) especially developed by ESA. The pretreatment procedure of GRD data includes radiometric correction, radiometric terrain flattening, speckle filtering using refined Lee filter with 7 × 7 windows [45], and geometric terrain correction with SRTM3 as a Digital Elevation Model (DEM). For SLC products, the preprocessing step includes polarization decomposition in addition to radiation correction, radiation terrain flattening, speckle filtering, and geometric terrain correction. Sentinel-2 optical images were atmospherically corrected with the Sen2Cor processor. Finally, Sentinel-1, Sentinel-2, and ground truth data were mapped to the WGS84 coordinate system, on which the ground control points were used to precisely match Sentinel-1 and Sentinel-2.

#### 3.3.1. Non-Polarization Decomposition Features

After the preprocessing of Sentinel multi-source data was completed, $\sigma_{VH}^{0}$, $\sigma_{VV}^{0}$, local incidence angle (LIA), and elevation could be directly extracted from Sentinel-1 GRD format data. The vegetation indexes that have been used in the previous literature were calculated by the red and near-infrared bands [23,28]. For Sentinel-2, B4 is red band, B8 is near-infrared band. The center wavelengths of B4 and B8 are 665 nm and 842 nm respectively, and their spatial resolutions are both 10 m (Table 3 shows more details).

**Figure 4:**
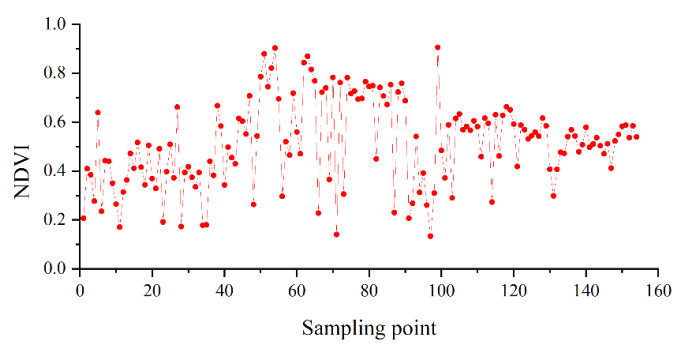
Normalized vegetation index (NDVI) values in sampling points.

For example, the NDVI values of sampling points were calculated and plotted in Figure 4; it can be seen that the NDVI values in 154 sampling points were obviously different.

#### 3.3.2. Polarization Decomposition Features

The H/A/*α* decomposition method was proposed by Cloude and Pottier based on the eigenvectors of the coherent matrix [49]. In fully PolSAR systems, the measured vector data can be expressed by the 2×2 complex scattering matrix as the following format,

(12) $$S_{0}=\left[\begin{array}{cc} S_{HH} & S_{HV}\\ S_{VH} & S_{VV} \end{array}\right]$$

where $S_{HH}$, $S_{HV}$, $S_{VH}$, and $S_{VV}$ are the scattering elements from four independent polarization channels, the subscripts “H” and “V” denote the horizontal and vertical linear polarizations, respectively. For the VV-VH polarization mode, the scattering matrix becomes

(13) $$S=\left[\begin{array}{cc} 0 & 0\\ S_{VH} & S_{VV} \end{array}\right].$$

And the target coherent matrix is decomposed into two parts through matrix features

(14) $$T=\sum_{i=1}^{2}\lambda_{i}e_{i}e_{i}^{T}.$$

Therefore, based on the quad-polarized data, the entropy (H), anisotropy (A), and alpha angle (*α*) are defined as (15), (16), and (17), respectively.

(15) $$H=\sum_{i=1}^{2}-P_{i}\log_{2}P_{i},\;P_{i}=\lambda_{i}/\sum_{i=1}^{2}\lambda_{i},\;0\leq H\leq 1$$

(16) $$A=\frac{\lambda_{1}+\lambda_{2}}{\lambda_{1}-\lambda_{2}},\;0\leq A\leq 1$$

(17) $$\alpha =\sum_{i=1}^{2}P_{i}\alpha_{i},\;0\leq \alpha \leq \frac{\pi }{2}.$$

It should be noted that the basic theory of H/*α* decomposition was originally proposed for the quad-PolSAR data, but the H/*α* decomposition could be modified for dual-polarization SAR; more detailed steps could be found in Reference [50]. The effect of SAR phase information on SMC retrieval would be indirectly judged by comparing the results with or without polarization decomposition features.

#### 3.3.3. Correlation Analysis between Input Parameters and SMC

**Figure 5:**
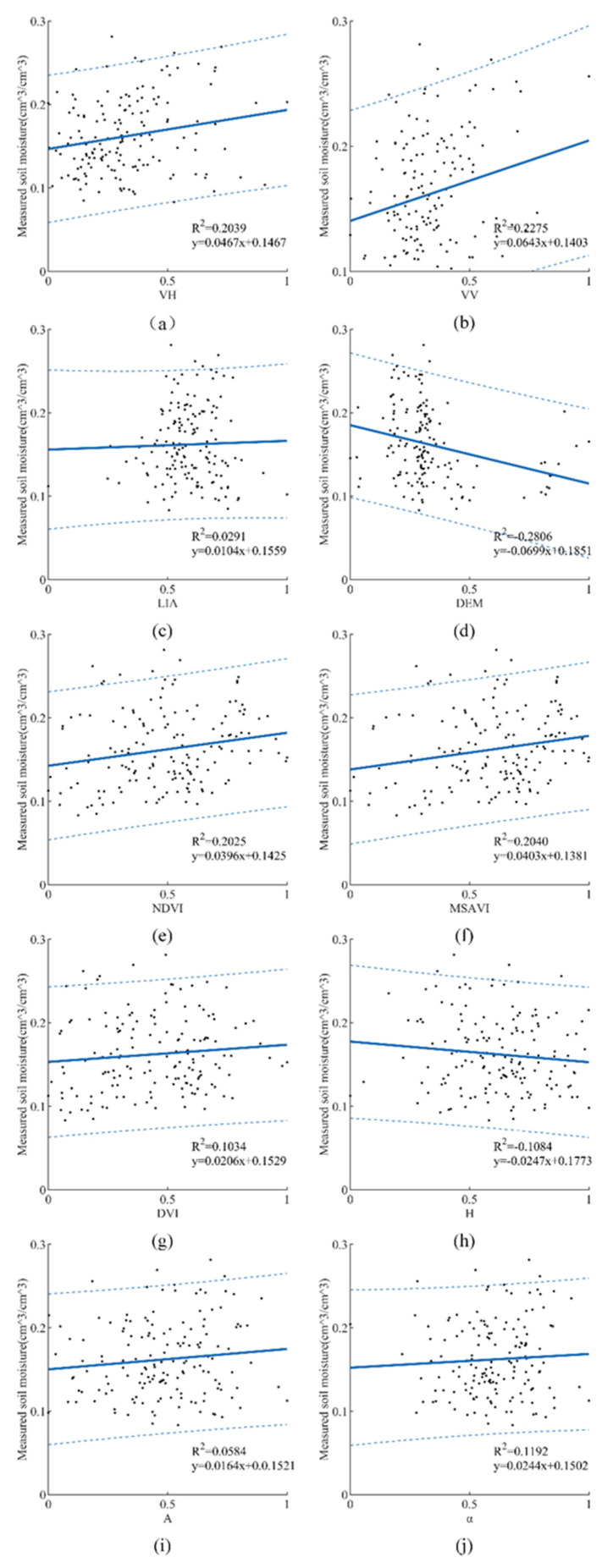
Correlation analysis between input parameters and SMC.

This part was to analyze the correlation between each feature parameter and SMC; the result is shown in Figure 5. It can be seen that VH, VV, and DEM have a certain correlation to SMC. However, the correlation between LIA and SMC is very weak. Among the three vegetation indexes, MSAVI has the strongest correlation with SMC, followed by NDVI, and DVI is the weakest. Among the three polarization decomposition variables, the correlation to SMC is *α*, H, and A in descending order.

## 4. Results

This section is organized as follows: firstly, the overall performance of the SVR, GRNN, and CNNR models was analyzed. Secondly, we investigated the potential of these retrieval models by comparing the results of six sets of feature parameter combinations. Finally, the relative importance of the feature parameters selected in the study was measured and determined by statistical methods. Data analysis and modeling are completed based on Matlab. In order to ensure the reliability of assessing soil moisture results, soil moisture evaluation statistics should be consistent with community standards [51], so the coefficient of determination (*R*^2^), root mean square error (RMSE), and mean relative error (MRE) were applied as three indicators for evaluating the accuracy.

### 4.1. Model Performances

**Figure 6:**
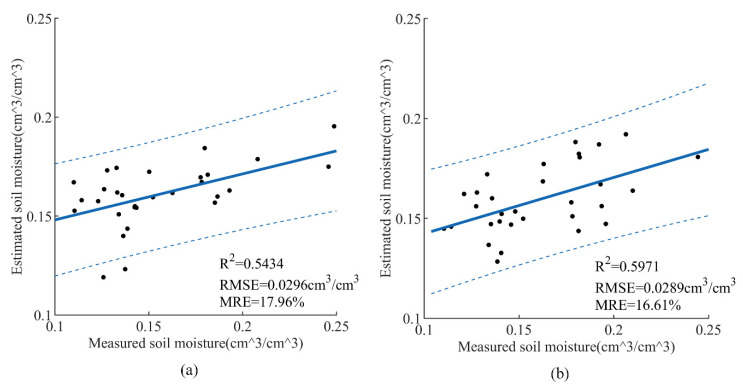
SMC retrieval results of the Oh model before and after removing vegetation: (**a**) before removing vegetation, (**b**) after removing vegetation.

SMC retrieval results of the Oh model before and after removing vegetation were demonstrated in Figure 6. The experimental results showed that the *R*^2^ of the test set increased by 0.0537 and the RMSE decreased by 0.0007 cm^3^/cm^3^ after removing the vegetation. Obviously, the accuracy of SMC retrieval had been improved after removing the vegetation by WCM.

**Figure 7:**
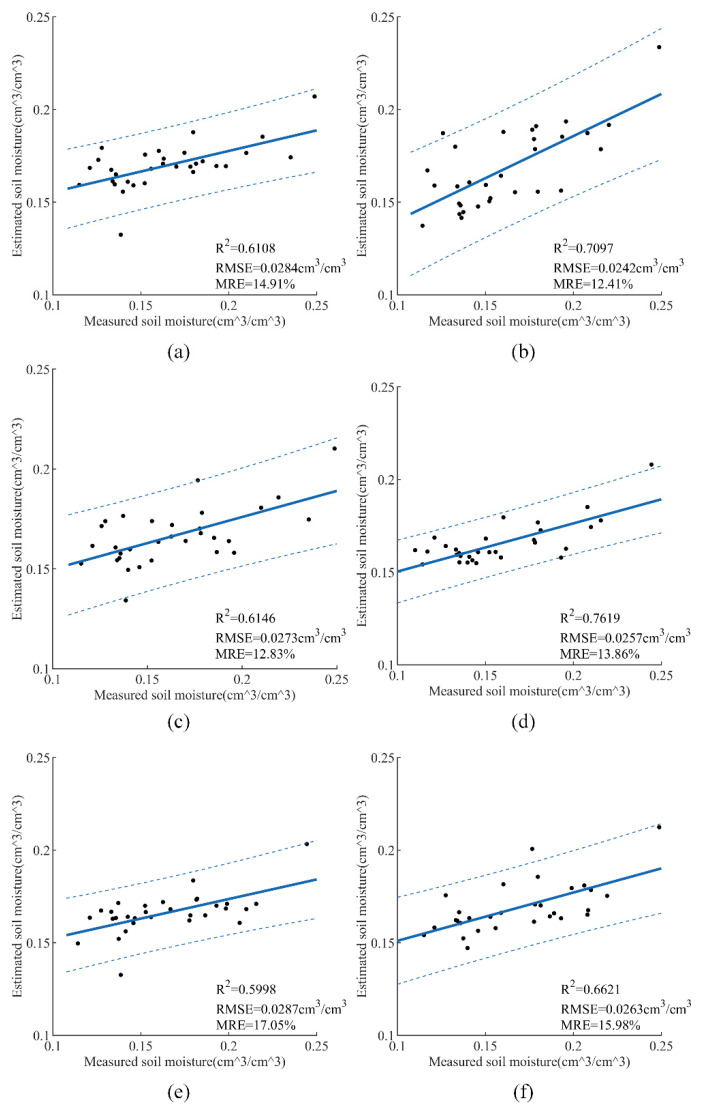
SMC retrieval results of the support vector regression (SVR) model with different feature combinations: (**a**) $\sigma_{VH}^{0}$ + $\sigma_{VV}^{0}$ + Digital Elevation Model (DEM) + local incidence angle (LIA) + NDVI, (**b**) $\sigma_{VH}^{0}$ + $\sigma_{VV\;}^{0}$ + DEM + LIA + NDVI + H + A + *α*, (**c**) $\sigma_{VH}^{0}$ + $\sigma_{VV}^{0}$ + DEM + LIA + MSAVI, (**d**) $\sigma_{VH\;}^{0}$ + $\sigma_{VV}^{0}$ + DEM + LIA + MSAVI + H + A + *α*, (**e**) $\sigma_{VH}^{0}$ + $\sigma_{VV}^{0}$ + DEM + LIA + DVI, (**f**) $\sigma_{VH}^{0}$ + $\sigma_{VV}^{0}$ + DEM + LIA + DVI + H + A + *α*.

First, we explored the prediction effect of the SVR model under different combinations that contain $\sigma_{VH}^{0}$, $\sigma_{VV}^{0}$, DEM, LIA, vegetation index, and polarization decomposition features. This model is trained and verified by six sets of feature parameter combinations. As can be seen from Figure 7a,c,e, when the input feature parameters are $\sigma_{VH}^{0}$ + $\sigma_{VV}^{0}$ + DEM+ LIA + NDVI/MSAVI/DVI, the *R*^2^ of the test set is 0.6108, 0.6146, and 0.5998 and the RMSE is 0.0284, 0.0273, and 0.0287 cm^3^/cm^3^, respectively. The results of Figure 7b,d,f indicate that the *R*^2^ of the test set is 0.7097, 0.7619, and 0.6621, and the RMSE is 0.0242, 0.0257, and 0.0263 cm^3^/cm^3^, respectively, after H, A, and *α* were added to the model input. Comparing the two groups of Figure 7a,c,e and Figure 7b,d,f, it can be obviously seen that the correlation between DVI and SMC is the weakest among the three vegetation indexes using the SVR model to estimate SMC.

**Figure 8:**
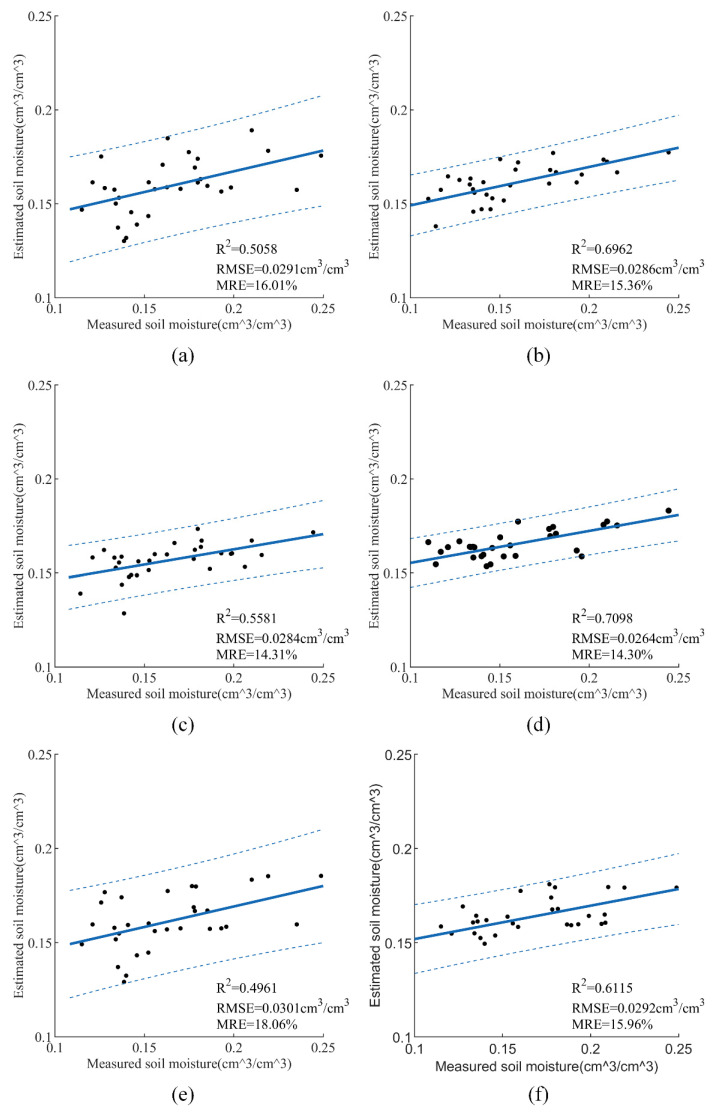
SMC retrieval results of the generalized regression neural network (GRNN) model with different feature combinations: (**a**) $\sigma_{VH}^{0}$ + $\sigma_{VV}^{0}$ + DEM + LIA + NDVI, (**b**) $\sigma_{VH}^{0}$ + $\sigma_{VV}^{0}$ + DEM + LIA + NDVI + H + A + *α*, (**c**) $\sigma_{VH}^{0}$ + $\sigma_{VV}^{0}$ + DEM + LIA + MSAVI, (**d**) $\sigma_{VH\;}^{0}$ + $\sigma_{VV\;}^{0}$ + DEM + LIA + MSAVI + H + A + *α*, (**e**) $\sigma_{VH}^{0}$ + $\sigma_{VV}^{0}$ + DEM + LIA + DVI, (**f**) $\sigma_{VH}^{0}$ + $\sigma_{VV}^{0}$ + DEM + LIA + DVI + H + A + *α*.

The same data processing procedure used in the previous SVR model was repeated in this stage to retrieve SMC based on GRNN. The resulting images were provided in Figure 8. As can be seen from Figure 8a,c,e, when the input feature parameters are $\sigma_{VH}^{0}$ + $\sigma_{VV\;}^{0}$ + DEM + LIA + NDVI/MSAVI/DVI, the experimental results indicated that the *R*^2^ is 0.5058, 0.5581, and 0.4964 and the RMSE is 0.0291, 0.0284, and 0.0301 cm^3^/cm^3^, respectively. The *R*^2^ is 0.6962, 0.7098, and 0.6115, and the RMSE is 0.0286, 0.0264, and 0.0292 cm^3^/cm^3^, respectively after H, A, and *α* were added to model input, as shown in Figure 8b,d,f. It could be concluded that adding polarization decomposition to input vectors results in better prediction performance. Comparing the two groups of Figure 8a,c,e and Figure 8b,d,f, we can obtain the similar conclusion that the correlation between DVI and SMC is the weakest among the three vegetation indexes using the GRNN model to retrieve SMC.

**Figure 9:**
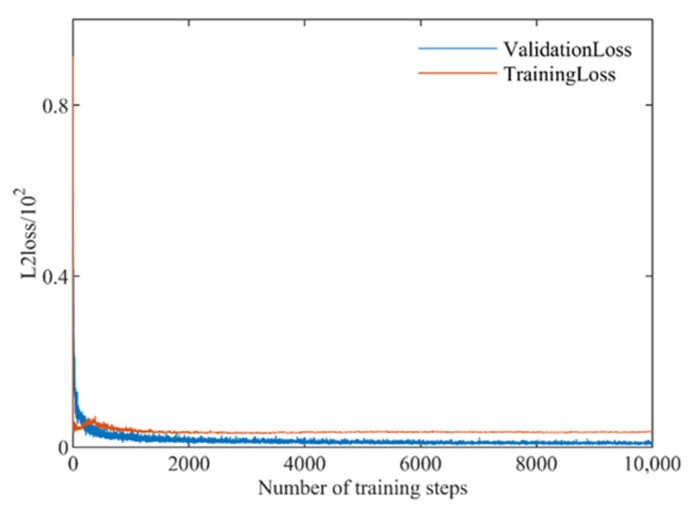
Loss function descent process.

A similar approach to construct an input vector in the previous SVR and GRNN model was applied for this CNN model. Figure 9 showed the change of loss function of the training and the validation set under feature combination ($\sigma_{VH}^{0}$ + $\sigma_{VV}^{0}$ + DEM + LIA + MSAVI + H + A + *α*). In the initial stage, the loss function value decreases rapidly. As the number of iterations increases, the network tends to converge, and the network has completely converged when the iteration ends, which indicated that the network is in a good learning state and there is no over-fitting.

**Figure 10:**
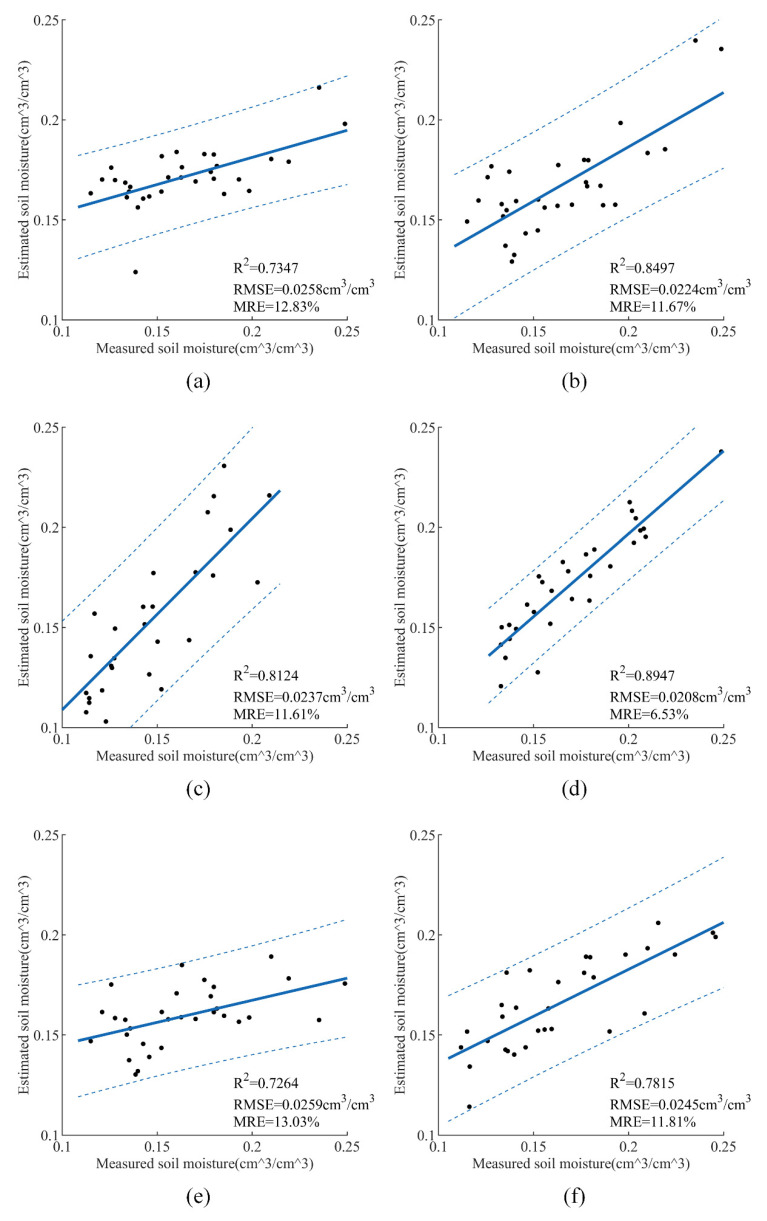
SMC retrieval results of the CNNR model with different feature combinations: (**a**) $\sigma_{VH}^{0}$ + $\sigma_{VV\;}^{0}$ + DEM + LIA + NDVI, (**b**) $\sigma_{VH}^{0}$ + $\sigma_{VV\;}^{0}$ + DEM + LIA + NDVI + H + A + *α*, (**c**) $\sigma_{VH}^{0}$ + $\sigma_{VV}^{0}$ + DEM + LIA + MSAVI, (**d**) $\sigma_{VH}^{0}$ + $\sigma_{VV}^{0}$ + DEM + LIA + MSAVI + H + A + *α*, (**e**) $\sigma_{VH\;}^{0}$ + $\sigma_{VV}^{0}$ + DEM + LIA + DVI, (**f**) $\sigma_{VH}^{0}$ + $\sigma_{VV}^{0}$ + DEM + LIA + DVI + H + A + *α*.

Figure 10 displays the results of validation set under different combinations. The *R*^2^ is 0.7347, 0.8124, and 0.7264 and the RMSE is 0.0258, 0.0237, and 0.0259 cm^3^/cm^3^, as Figure 10a,c,e shows. The *R*^2^ of inputs with the addition of polarization decomposition features is 0.8497, 0.8947, and 0.7815, and the RMSE is 0.0224, 0.0208, and 0.0245 cm^3^/cm^3^, respectively. Furthermore, we could find that the linear relationship between DVI and SMC is the weakest among the selected vegetation indices.

### 4.2. Analysis of Model Inversion Results

It can be seen from the above results that the feature combination ($\sigma_{VH}^{0}$ + $\sigma_{VV\;}^{0}$ + DEM + LIA + MSAVI + H + A + *α*) is the optimal combination of the SVR, GRNN, and CNNR models. The optimal combination of *R*^2^ and RMSE of SVR is 0.7619 and 0.0257 cm^3^/cm^3^, respectively. The optimal combination of *R*^2^ and RMSE of the GRNN is 0.7098 and 0.0264 cm^3^/cm^3^, respectively. The optimal combination of *R*^2^ and RMSE of the CNNR is 0.8947 and 0.0208 cm^3^/cm^3^, respectively.

Moreover, it is necessary to analyze the influence of polarization decomposition features on SMC. For SVR models, the *R*^2^ of inputs with the addition of polarization decomposition features was increased by 0.0989, 0.1473, and 0.0623, and the RMSE was decreased by 0.0042, 0.0016, and 0.0024 cm^3^/cm^3^, respectively in comparison to the previous input of five characteristic parameters. For GRNN models, the *R*^2^ of inputs with the addition of polarization decomposition features was increased by 0.1904, 0.1517, and 0.1151, and the RMSE was decreased by 0.0005, 0.0020, and 0.0009 cm^3^/cm^3^, respectively. For CNNR models, the *R*^2^ of inputs with the addition of polarization decomposition features was increased by 0.1904, 0.1517, and 0.1151, and the RMSE was decreased by 0.0005, 0.0020, and 0.0009 cm^3^/cm^3^, respectively. According to the statistics of the six groups under SVR, GRNN, and CNNR models, the RMSE of the model decreases after adding the H, A, and *α* parameters, which means that these decomposition features have a positive effect on the SMC retrieval. The polarization decomposition features contain information different from the radar backscattering coefficient, which can reflect the texture and roughness of the target object. Therefore, these models performed better after the polarization decomposition features were added.

Compared with the results in Figure 5, the accuracies of SMC retrieval were significantly improved when input features were combined. Remote sensing data are affected by factors such as SMC, vegetation, and radar incidence angle, so richer information is helpful to establish better correspondence. For the Oh model, the SMC retrieval accuracy was raised after removing the vegetation influence. For SVR, GRNN, and CRNN, the kernel function was used to directly couple the relationship between SMC and remote sensing parameters. In this case, CNNR had the strongest ability to extract features from the remote sensing data; thus, its accuracy was the highest. In summary, the key to constructing an SMC retrieval model is to obtain features related to SMC.

### 4.3. Analysis of Feature Parameters Importance

The SMC prediction results are not only influenced by the inversion model but also by the different combination of characteristic parameters, so it is necessary to further analyze the effect of each characteristic parameter on the SMC inversion results. Each possible combination is composed of $\sigma_{VH}^{0}$, $\sigma_{VV}^{0}$, DEM, LIA, H, A, and *α* and vegetation indexes. The seven inputs ($\sigma_{VH}^{0}$, $\sigma_{VV}^{0}$, DEM, LIA, H, A, *α*) give 27 combinations, one vegetation index is involved at most among the three vegetation indexes in each input combination (none, NDVI, MSAVI, DVI), so the total combinations are 27 × 4 = 512 (combinations = [$\sigma_{VH}^{0},\cdot \cdot \cdot ,\sigma_{VH}^{0}+\mathrm{LIA},\cdot \cdot \cdot ,\sigma_{VH}^{0}+H+\mathrm{NDVI},\cdot \cdot \cdot ,\;\sigma_{VH}^{0}$ + $\sigma_{VV}^{0}$ + DEM + LIA + H + A + *α* + MSAVI]). Furthermore, this study defines the equivalent number to analyze the relative importance of each feature parameter more objectively (Formula (18)).

(18) $$EN=\frac{RMSE_{average}}{RMSE_{chosen}}$$

where *EN* means equivalent number, $RMSE_{average}$ and $RMSE_{chosen}$ are the average root mean square error of all selected combinations and the root mean square error of a certain combination, respectively.

**Figure 11:**
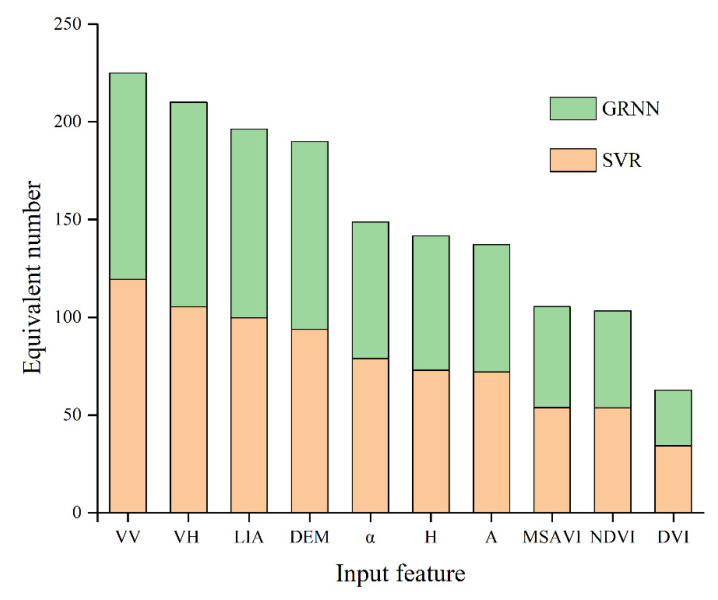
Equivalent number of input features.

The equivalent number of each input feature was calculated by a partial combination of SVR and GRNN models, where the RMSE of the test set of these combinations was required to be less than 0.035 cm^3^/cm^3^, and the *R*^2^ was greater than 0.5. After screening, 160 groups in SVR and 146 groups in GRNN were used to calculate the equivalent number. The counting results are shown in Figure 11.

Although the equivalent number of each input feature in the two models are different, the appearance trend is the same, from large to small arranged as $\sigma_{VH}^{0}$, $\sigma_{VV}^{0}$, LIA, DEM, MSAVI, NDVI, and DVI. Among these features, $\sigma_{VV}^{0}$ (225.01/306) was more frequent than $\sigma_{VH\;}^{0}$ (210.17/306), which is consistent with the conclusion made by Mirsoleimani et al. [14] that $\sigma_{VV}^{0}$ was more sensitive to SMC than $\sigma_{VH}^{0}$. The correlation analysis between input parameters and SMC in Figure 5 varied that $\;\sigma_{VV}^{0}$ was more related to SMC than $\sigma_{VH}^{0}$, too. This was mainly because the co-polarized VV backscattering mode contains richer soil scattering information and reflects more surface information than the cross-polarized VH backscattering mode. Altitude DEM (189.96/306) was supported by the fact that Holtgrave et al. [18] verified that altitude was an important feature of SMC retrieval. Among the three vegetation indexes, MSAVI (105.45/306) has the strongest correlation with SMC, which is followed by NDVI (103.26/306) and DVI (62.72/306), as described by Figure 11. Meanwhile, the results in Figure 5 also indicated that the correlation to SMC is MSAVI, NDVI, and DVI in descending order. This may be caused by the fact that MSAVI simultaneously presents the influences of both vegetation and soil background, which makes its response to SMC change more significantly in the area.

## 5. Discussion

### 5.1. Contribution of Polarization Decomposition Features

On the one hand, Figure 11 indicated that H, A, and *α* were all sensitive to SMC. Judging from the equivalent number of the three parameters, the counting results of H (141.68/306) and A (137.22/306) are relatively close, and *α* (148.81/306) led with a slight advantage. The statistical results led to the conclusion that the addition of polarimetric decomposition features is of superiority in SMC retrieval. This is supported by the fact that Özerdem et al. found that H, A, and *α* are sensitive with SMC [19].

On the other hand, after mixing H, A, and *α* features, the CNNR models were able to estimate SMC with RMSE between 0.0208 and 0.0245 cm^3^/cm^3^ and *R*^2^ between 0.78 and 0.89. To further investigate the quantitative impact of H, A, and *α*, the comparison result of SVR, GRNN, and CNNR under six selected combinations is listed in Table 4. Compared to non-polarization decomposition feature combinations, the average *R*^2^ and RMSE values of SVR, GRNN, and CNNR with polarization decomposition feature combination were obviously better. Through further calculating, we could conclude that the *R*^2^ of SVR, GRNN, and CNNR increased by 0.1028, 0.1524, and 0.1524, respectively, and the RMSE of SVR, GRNN, and CNNR decreased by 0.0027, 0.0012, and 0.0019 cm^3^/cm^3^, respectively. Undoubtedly, the polarization decomposition features are beneficial to raising the accuracy of SMC retrieval.

### 5.2. Differences from Existing Work

During this paper, different input features in all possible combinations were analyzed to confirm and consider the main features applicable to farmland areas. It is difficult to effectively estimate vegetation in the process of inversing farmland surface SMC using remote sensing data. Different from other literatures considering the effect of vegetation cover on SMC [25,26], one contribution of this paper is that it applied vegetation index and polarization decomposition features to characterize vegetation contribution. Among the three indexes, MSAVI shows good linear correlation with SMC. After adding the polarization decomposition feature to the input vector, the accuracy had been improved, so it is possible to consider SAR phase information when retrieving SMC in the future. Overall, when the model input has sufficient parameters, statistical results display that these algorithms show good performance and prediction effects, especially the CNNR model.

Meanwhile, this paper defined an equivalent number to evaluate the relative importance of each characteristic parameter, which can better reflect the differences between groups, so it is more objective and reasonable than simply counting. The results verify the important effects of the radar backscatter coefficient, altitude, local incidence angle, and vegetation index on the retrieval of farmland surface SMC by counting its equivalent number.

Another contribution of this paper was that regression convolutional neural networks were constructed to SMC retrieval with the fusion of SAR and optical data. This proposed CNNR model could fully consider the spatial distribution of features and extract advanced features reflecting the spatial and temporal differences of SMC. Meanwhile, the pooling layer commonly used in the traditional convolutional neural network structure was removed, which not only ensured the integrity of the extracted feature information but also accelerated the network training and predicting speed. In addition, the method employed does not require evaluating the impact of vegetation, thus avoiding errors in the process of estimating vegetation.

Furthermore, in the same dataset, this CNNR model could better fuse satellite data of different observation modes and types, which made the utilization of satellite images more efficient. Test results confirmed that CNNR has higher applicability to retrieve SMC in our experimental area than the traditional Oh model, SVR, and the GRNN model.

The results proved that merging the intensity information and phase information of SAR data can effectively raise the accuracy of SMC. Under similar scenarios and data sources, the overall accuracy of the proposed approach shows good performance in comparison to other methods in the literature, and some main methods for retrieving SMC are listed in Table 5.

### 5.3. Limitations and Future Work

Firstly, since the Sentinel-1 data used in this paper were limited to dual-polarized data, only the H/A/*α* decomposition model was employed to investigate and verify the influences of SAR phase information to SMC. Actually, there have been several polarimetric decomposition techniques used in inverting SMC, so further research could be carried out together with other common polarization decomposition methods to improve the retrieval accuracy. Secondly, vegetation indices vary with plant growth stage and vegetation type, and the original band that involved better versatility and consistency than vegetation indices could be applied in future work. Thirdly, although Sentinel-1 SAR imaging is not affected by environmental factors such as clouds and fog, the red and near-infrared bands used in this model are sensitive to weather. Thus, the application of this method is occasionally restricted by the availability of Sentinel-2 optical imagery.

## 6. Conclusions

This paper aimed at evaluating the capability of using Sentinel multi-source data to retrieve SMC over farmland areas by regression convolutional neural networks. In our proposed approach, the H/A/*α* decomposition method was firstly implemented to extract polarization decomposition features that are merged with other feature parameters to form the input vector. Then various retrieval methods were employed to estimate surface SMC.

The experimental results confirmed that vegetation index and polarization decomposition features could characterize vegetation contribution effectively over farmland areas. The addition of polarimetric decomposition features is effective to improve the accuracy of soil moisture retrieval results. Among these retrieval models, CNNR performed the best compared to the traditional Oh model, SVR, and GRNN, whose *R*^2^ and RMSE reach up to 0.8947 and 0.0208 cm^3^/cm^3^, respectively. These results indicated that CNNR has great potential for estimating soil moisture from Sentinel multi-source data and could provide good prospects for local farmland irrigation and water conservation.

## Figures and Tables

**Table 1:** Collected soil moisture content (SMC) data in the study areas.

Date	Number of Samples	Min–Mean–Max SMC (cm^3^/cm^3^)	SD of SMC
12 March 2018	45	0.0956–0.1624–0.2618	0.0398
8 December 2019	54	0.0832–0.1761–0.2692	0.0473
6 January 2020	55	0.0850–0.1478–0.2814	0.0427

**Table 2:** Characteristics of Sentinel-1 and Sentinel-2 data used in this study.

Satellite Platform	Acquisition Dates	Data Modes	Data Level	Polarizations or Bands	Time Resolution	Spatial Resolution
Sentienl-1A	12 March 2018	GRD + SLC	Level 1	VH + VV	12 days	5 m × 20 m
	8 December 2019					
	6 January 2020					
Sentinel-2A	10 March 2018	MSI	Level 1C	Red + Near Infrared	10 days	10 m × 10 m
	8 December 2019					
	6 January 2020					

**Table 3:** Adopted vegetation indices calculated from Sentinel-2 data.

Vegetation Index (VI)	Index Full Name	Formulae	References
NDVI	Normalized Difference Vegetation Index	$NDVI=(B_{8}-B_{4})/(B_{8}+B_{4})$	[46]
DVI	Difference Vegetation Index	$DVI=B_{8}-B_{4}$	[47]
MSAVI	Modified Soil Adjusted Vegetation Index	$MSAVI=\frac{1}{2}[(2B_{8}+1)-\sqrt{\left(2B_{8}+1)-8(B_{8}-B_{4}\right)}]$	[48]

**Table 4:** Comparison of the average of SVR, GRNN, and CNN under six selected combinations.

Model	Non-Polarization Decomposition Feature Combination			Polarization Decomposition Feature Combination		
	*R* ^2^	RMSE	MRE	*R* ^2^	RMSE	MRE
SVR	0.6084	0.0281	14.93%	0.7112	0.0254	14.08%
GRNN	0.5201	0.0292	16.13%	0.6725	0.0280	15.12%
CNNR	0.7578	0.0251	12.48%	0.8419	0.0232	10.02%

**Table 5:** Comparison of different approaches for estimating SMC.

Authors	Data	Accuracy	Methods
**SMC retrieval over bare areas**			
Paloscia [16]	Sentinel-1	*R*^2^ = [0.59–0.88]	ANN
Balenzano [21]	Airborne SAR	*R*^2^ = [0.5–0.7]	Change detection technique
Hajnsek [52]	POLSAR	*R*^2^ = [0.4–0.7]	Polarimetric decomposition
Hachani [53]	Sentinel-1	*R*^2^ = 0.77	ANN
Zribi [54]	ALOS-2	RMSE = [6.7–16.1%]	Backscattering model
Pierdicca [55]	AirSAR	*R*^2^ = [0.59–0.79]	Bayesian method
Satalino [56]	ERS-SAR	RMSE = [3.01–3.14%]	MLP and IEM
**SMC retrieval over vegetated areas**			
Baghdadi [23]	Sentinel-1/2; Landsat-8	RMSE = 6%	NNs
Kong [25]	Radarsat-2/GF-1	*R*^2^ = [0.82–0.87]	AIEM and WCM
Attarzadeh [28]	Sentinel-1/2	RMSE = [4.94–6.41%]	SVR
Mattia [57]	Sentinel-1/2	*R*^2^ = 0.5	Change detection technique
Han [58]	GF-3/GF-1	RMSE = [0.0271–0.0321 cm^3^/cm^3^]	Optimal solution method
Adab [59]	SMAP/Landsat 8	*R*^2^ = 0.73	Machine learning
Wang [60]	Sentinel-1/2; Landsat-8; GF-1	*R*^2^ = [0.51–0.623]	AIEM and LUT
Proposed Method	Sentinel-1/2	*R*^2^ = [0.72–0.89] and RMSE = [0.0208–0.0259 cm^3^/cm^3^]	CNN

## Data Availability

Data is contained within the article.
